# Doctor's perception of doctor-patient relationships in emergency departments: What roles do gender and ethnicity play?

**DOI:** 10.1186/1472-6963-8-82

**Published:** 2008-04-11

**Authors:** Birgit Babitsch, Tanja Braun, Theda Borde, Matthias David

**Affiliations:** 1 Berlin Institute of Gender in Medicine (GiM), Charité – Universitätsmedizin Berlin, Luisenstr. 65, 10117 Berlin, Germany; 2 County Durham Primary Care Trust, Fern Court, Bracken Hill Business Park, Peterlee, SR8 2RR, UK; 3 Alice Salomon University of Applied Science, Alice-Salomon-Platz 5, 12627 Berlin, Germany; 4 Clinic for Gynaecology and Obstetrics, Charité – Universitätsmedizin Berlin, Augustenburgerplatz 1, 13353 Berlin, Germany

## Abstract

**Background:**

Emergency departments continuously provide medical treatment on a walk-in basis. Several studies investigated the patient's perception of the doctor-patient relationship, but few have asked doctors about their views. Furthermore, the influence of the patient's ethnicity and gender on the doctor's perception remains largely unanswered.

**Methods:**

Based on data collated in three gynaecology (GYN)/internal medicine (INT) emergency departments in Berlin, Germany, we evaluated the impact of the patient's gender and ethnicity on the doctors' satisfaction with the course of the treatment they provided. Information was gathered from 2.429 short questionnaires completed by doctors and the medical records of the corresponding patients.

**Results:**

The patient's ethnicity had a significant impact on the doctors' satisfaction with the doctor-patient relationship. Logistic regression analysis showed that the odds ratio (OR) for physician satisfaction was significantly lower for patients of Turkish origin (OR = 2.6 INT and 5.5 GYN) than for those of German origin. The main reasons stated were problems with communication and a perceived lack of urgency for emergency treatment. The odds ratios for dissatisfaction due to a lack of language skills were 4.48 (INT) and 6.22 (GYN), and those due to perceived lack of urgency for emergency treatment were 0.75 (INT) and 0.63 (GYN). Sex differences caused minor variation.

**Conclusion:**

The results show that good communication despite language barriers is crucial in providing medical care that is satisfactory to both patient and doctors, especially in emergency situations. Therefore the use of professional interpreters for improved communication and the training of medical staff for improved intercultural competence are essential for the provision of adequate health care in a multicultural setting.

## Background

Emergency departments provide health care to severely ill and emergency patients. A crucial difference to many other health care services is that they are available for 24-hours, 7 days a week on a walk-in basis. In Germany, emergency outpatient treatment is provided by public and private hospitals with emergency departments. The utilisation of emergency services has risen worldwide [1,2] due demographic changes and increasing demand [1]. In Germany, emergency departments appear to be an access point for patients who cannot easily access other health care services [3]. Of the few German studies conducted so far, most show a disproportionately high use of emergency facilities by immigrants compared to native Germans [4,5].

Emergency doctors are subject to particularly high levels of work related stress. Causes are a high demand for treatment, time pressures and the organisational structure of the German health care system where emergency departments are the junction between primary and secondary care. Additionally, the work of emergency doctors is demanding because of patients with a wide range of problems and needs and of the urgency of many problems. Patients utilising emergency facilities feel an urgent need for treatment and expect immediate and adequate care. These perceptions have a direct impact on the demand for health care services particularily emergency services and can easily lead to organisational problems such as long waiting times.

Very few studies have investigated the satisfaction of emergency doctors with the treatment they provide [6,7] and have looked at related influence variables. So far, no study has analysed the impact of gender and/or ethnicity on the satisfaction of emergency doctors with treatment. The present study was aimed to elucidate the following questions:

1. To what extent is the emergency doctor's satisfaction with the course of treatment influenced by the patient's sex and ethnicity?

2. Which other factors lead to sex and ethnicity-specific differences in the doctor's satisfaction with the course of the treatment?

We hypothesised that sex and ethnicity of the patients have an independent effect on the doctor's satisfaction with the treatment provided. Other factors investigated include communication competence (especially language skills), the perceived urgency for emergency treatment, and the time of the day when the patient arrived at the emergency department. We expected that these factors would be partly responsible for any observed sex and ethnicity differences.

This study, which focuses on emergency service providers and variables that affect their satisfaction with doctor-patient relationships, can contribute significantly to the current research on doctor-patient relationship as well as on the patient's satisfaction.

## Methods

The study took place between November 2001 and April 2002 in three emergency departments for internal medicine and gynaecology in Berlin, Germany. Approval was given by the ethics committee of the Charité – Universitätsmedizin Berlin. The hospitals (Charité – Campus Virchow-Klinikum, Vivantes Klinikum am Urban, Vivantes Klinikum Neukölln) were selected because of their location in districts with a high proportion of immigrants. All hospitals are located in socially disadvantaged districts of Berlin with a lower educational status and a high rate of unemployment.

First, data from 2969 emergency consultations (50.6% of all treatments) were collected using a short questionnaire, which was to be completed by the doctors (N = 80) immediately after treatment. Questionnaire items surveyed included: the doctor's perception of the urgency for emergency treatment, the native language of the patient, the language of the patient-doctor communication, the need for and use of interpreters, and the perceived quality of the doctor-patient relationship. Secondly, the corresponding patient medical data set, which included medical case history, examination, results, diagnosis, and treatment, was gathered from the patients' medical records. Altogether at each hospital for a full month, 5872 patient records were screened. Only information from patients with German or Turkish origin, the two largest sub-groups, was further analysed. Information from patients with different other ethnic backgrounds was excluded either because the groups were very small or the heterogenity of many ethnic groups vary greatly with respect to place of origin, native language and domestic context, which make it difficult to combine them into a single group (see detailed [5]). Finally, a total of 2429 data sets were analysed.

### Variables

The dependent variable was the doctors' satisfaction with the course of the treatment, which was rated using a 5-grade Likert scale (very satisfactory to very unsatisfactory). Doctors who rated the course of treatment as unsatisfactory or very unsatisfactory were asked to specify the cause for their dissatisfaction. The responses were retrospectively classified into nine main categories. Likert scale responses were classified as "satisfied" (1–2) or "dissatisfied" (3–5).

Sex and ethnicity were the primary independent variables. Ethnic origin was determined based on the native language of the patient if specified, or by the name of the patient if not. In latter case three investigators with a different ethnic background (Turkish, Kurdish, and German), performed a name analysis independently using the patients' Christian and last name. In indecisive cases investigators with other ethnic backgrounds were involved and in cases of mismatch the ethnicity were kept as unknown. Especially in the Turkish population the method of name analysis has proved to be a valid approach [6].

Other variables included: utilisation characteristics (time of presentation to the emergency department, means of transportation, time since onset of complaints, urgency for emergency treatment as perceived by the doctor), patient characteristics (age, sex, postcode, health insurance company as a proxy for social status), doctor's characteristics (professional experience, sex), and aspects of the doctor-patient communication (utilisation of interpreters, quality and language of communication as a proxy for language skills, length of medical history). The quality of the communication was also graded on a Likert scale using two categories: "good" (1–2) and "poor" (3–5). The perceived urgency of the treatment was rated by the attending doctors on a Likert scale from low (1) to high (10).

### Statistical analysis

All groups were stratified by sex and ethnicity as well as by type of speciality (INT or GYN). Patients were further divided into four subgroups: German women (GW), German men (GM), Turkish women (TW) and Turkish men (TM). The analysis was performed using the statistical package SPSS 14.0^® ^for Windows.

Socio-demographic information about patients and doctors as well as selected data describing the utilisation of services were cross-tabulated by sex for patients and doctors, and by ethnicity for patients. Pearson's Chi^2 ^test, Mann-Whitney U-test and the Kruskal-Wallis H-test were used to test for significant differences between the subgroups. The level of significance was set to p ≤ 0.05 for all statistical tests. Bonferroni correction was applied as needed. Correlation analyses were performed using the Spearman r and Pearson r.

Logistic regression analysis was used to test for dependence of the doctors' satisfaction with the course of treatment with the patient's sex and ethnicity. Two models were developed: the first model analysed the influence of the patients' sex and ethnicity, the second model looked at the patients' sex and ethnicity as well as communication competence and the urgency for emergency treatment as perceived by the doctors. A third analysis, which was performed as a stepwise forward logistic regression, analysed patient's characteristics (age, sex, ethnicity, utilisation of interpreters, type of health insurance), facility use characteristics (time of presentation, means of transportation, duration of complaints, need for emergency treatment as perceived by the doctor, administration or prescription of medication); doctor's characteristics (professional experience, sex), and aspects of doctor-patient communication (communication competence, length of medical history). Dummy variables were created if necessary. The inclusion criterion was set to p ≤ 0.05.

## Results

### Socio-demographic characteristics of physicians and patients

A total of 28.9% of INT doctors and 73.1% of GYN doctors in the emergency departments were female. Differences regarding the professional experience were found among both, female and male doctors as well as between the respective specialities (Table 1). INT female doctors were in 39.5% junior house officers, 17.0% senior house officer, 26.2% registrars and 15.2% consultants compared to 13.0% junior house officers, 36.9% senior house officer, 48.4% registrars and 1.8% consultants among male doctors. Similar differences appeared in the GYN clinics. The professional experience of INT versus GYN doctors was 20.2% vs. 14.9% junior house officers, 29.8% vs. 62.5% senior house officer, 40.2% vs. 18.1% registrars, and 5.6% vs. 1.3% consultants.

**Table 1:** Doctor's sex and occupational status

	Internal Medicine^1)^			Gynaecology^2)^		
	Women (n = 610)	Men (n = 1.420)	All (n = 2.109)	Women (n = 234)	Men (n = 74)	All (n = 320)

Occupational status (%)						
Junior house officers	39.5	13.0	20.2	17.1	8.1	14.9
Senior house officer	17.0	36.9	29.8	58.1	86.5	62.5
Registrars	26.2	48.4	40.2	24.8	0	18.1
Consultants	15.2	1.8	5.6	0	5.4	1.3

^1) ^Chi^2 ^= 384.6, df = 3, p < .000, ^2) ^Chi^2 ^= 41, df = 3, p < .00, Mann-Whitney-U-Test Internal Medicine: p < .001; missing values are not shown.

The patients differed significantly in respect to sex and ethnicity (see Table 2). The proportion of patients of German origin was higher than of patients with Turkish origin. German patients were older. Both results reflect the proportion of immigrants and the age distribution of the German and Turkish populations in Berlin [4]. Most of the patients were members of one of the statutory or private health insurance companies. The treatment costs of a rather small percentage of 9.2% of German males, 7% of Turkish males and females, and only 3.6% of German women were covered by the welfare office.

**Table 2:** Socio-demographic characteristics of the patients

	Internal Medicine					Gynaecology		
	GW (n = 856)	TW (n = 698)	GM (n = 273)	TM (n = 282)	p^1)^	GW (n = 195)	TW (n = 125)	p^1)^

Age groups (%)					***			**
15 to 29 years	19.3	39.9	11.0	25.2		46.2	61.6	
30 to 49 years	21.8	31.9	31.9	44.7		39.5	32.8	
50 to 64 years	17.5	23.1	24.5	20.9		6.7	5.6	
≥ 65 years	41.2	4.8	32.5	9.2		6.2	0	
Health insurance (%)					***			n.s.
Compulsory health insurance	93.1	90.5	84.2	89.0		92.8	92.8	
Private health insurance	2.1	1.5	5.2	1.4		2.6	3.2	
Coverage through welfare	3.6	7.0	9.2	7.1		2.1	3.2	
Other	1.2	1.1	1.4	2.5		2.6	0.8	

GW = German women, GM = German men, TW = Turkish women, TM = Turkish men; ^1)^Kruskal-Wallis test: ** p ≤ 0.01. *** p ≤ 0.001, n.s.: non-significant

### Health condition of patients

Table 3 and 4 show the ICD10-diagnosis of INT and GYN patients with a prevalence higher than 5%. INT patients differed significantly regarding the diagnosis (see Table 3). Diseases of the circulatory system were the most important condition in German patients (24.1%) and in Turkish men (18.2%). 21.7% in Turkish women were diagnosed with Symptoms, signs and abnormal clinical and laboratory findings, not elsewhere classified. The second most frequent diagnosis for German women were diseases of the digestive system (14.8%), in Turkish women diseases of the circulatory system (16.8), in German men diseases of respiratory system (14.2%), and in Turkish men mental and behavioural disorders (14.4%). GYN patients were most frequently diagnosed with pregnancy, childbirth and the puerperium or diseases of the genitourinary system (see Table 4). The differences between German and Turkish patients were not significant.

**Table 3:** Diagnosis of the patients – Internal Medicine

	GW (n = 856)	TW (n = 698)	GM (n = 273)	TM (n= 282)
**ICD 10 Diagnostic Groups **(%)				
Mental and behavioural disorders	6,0	9,4	9,5	14,4
Diseases of the nervous system	4,7	3,5	5,1	4,5
Diseases of the circulatory system	24,1	16,8	24,1	18,2
Diseases of the respiratory system	13,2	14,3	14,2	12,6
Diseases of the digestive system	14,8	11,7	12,9	14,2
Diseases of the genitourinary system	6,0	5,8	3,7	4,2
Symptoms, signs and abnormal clinical and laboratory findings, not elsewhere classified	12,3	21,7	11,5	16,5

GW = German women, GM = German men, TW = Turkish women, TM = Turkish men; Kruskal-Wallis test: p ≤ 0.001

**Table 4:** Diagnosis of the patients – Gynaecology

	GW (n = 195)	TW (n = 125)
**ICD 10 Diagnostic Groups **(%)		
Pregnancy, childbirth and the puerperium	34,7	42,3
Diseases of the genitourinary system	30,5	28,5
Symptoms, signs and abnormal clinical and laboratory findings, not elsewhere classified	12,1	15,4
Neoplasms	5,3	0
Others	8,9	1,6

GW = German women, TW = Turkish women, Kruskal-Wallis test: n.s.

### Utilisation of emergency outpatient services: The role of the patient sex and ethnicity

Service utilisation differed significantly according to sex and ethnicity (Table 5). The longest interval between the onset of complaints and the utilisation of emergency services was observed in German men, and the shortest in German women. Use of private transportation was more typical for Turkish patients (men: 74.1%; women 81.0%) than for German patients (men: 51.6%; women 49.9%). Roughly one-third of all German patients reached the INT facilities via mobile intensive care units compared to 12.8% of Turkish females and 15.6% of Turkish males. These differences were smaller in the GYN groups.

**Table 5:** Emergency department utilisation characteristics

	Internal Medicine					Gynaecology		
	GW (n = 856)	TW (n = 698)	GM (n = 273)	TM (n = 282)	p^1)^	GW (n = 195)	TW (n = 125)	p^1)^

Time since onset of complaints					n.s.			n.s.
Mean (hours)	84	121	157	123		78	119	
Standard deviation	193	378	1041	490		186	390	
Transportation (%)					***			n.s.
Private	49.9	81.0	51.6	74.1		88.2	92.8	
General ambulance service	15.5	4.0	10.6	6.4		3.1	1.6	
Mobile intensive care unit	29.2	12.8	34.1	15.6		5.6	2.4	
Emergency ambulance	5.0	1.5	2.4	3.9		0	0	
Police	0.1	0	1.3	0		0	0	
Time of presentation (%)								
Time of day (%)					***			n.s.
8 am – 8 pm	70.6	56.0	68.8	49.3		73.3	62.4	
8 pm – 8 am	29.1	43.6	31.2	50.7		26.7	37.6	
Day of week (%)					***			n.s.
Weekday	66.2	53.8	69.1	55.3		58.5	56.0	
Weekend	33.8	46.2	30.9	44.7		41.5	44.0	
Need for emergency treatment as perceived by physician					***			n.s.
Mean	6	5	6	5		5	5	
Standard deviation	3	2	3	3		4	3	
Need for admission (%)					***			n.s.
Yes	37.9	14.3	42.1	23.0		17.9	12.8	
Medication given or prescribed					***			n.s.
Yes	47,3	54,8	42,1	50,2		43,4	44,9	
Need for translation (%)					***			***
Yes	0.5	36.3	0	34.6		1.1	36.3	
Interpreter^2) ^(%)					***			***
Accompanying relative/friend	0.4	32.6	0	24.8		1.0	33.6	
Nurse	0.1	0.7	0	0		0	0	
Physician	0	0.4	0	0.4		0	2.4	
Other hospital personnel	0	0.7	0	0.4		0	0	
Length of medical history (number of words)					n.s.			n.s.
Mean	13	12	12	11		12	14	
Standard deviation	9	7	8	7		8	8	

GW = German women, GM= German men, TW = Turkish women, TM = Turkish men; ^1)^Kruskal-Wallis test; ** p ≤ 0.01, *** p ≤ 0.001; ^2)^Differences in percentages results from missing values.

The doctor's perception of urgency for emergency treatment was significantly lower for Turkish INT patients compared with German INT patients. The time of the day when patients arrived at the emergency department differed greatly between German and Turkish patients. Turkish patients consulted the services more frequently out-of-hours (on weekends and between 8 p.m. and 8 a.m.) than German patients. German INT patients were more often admitted to inpatient treatment than their Turkish counterparts, while there was no significant difference for inpatient admissions in the gynaecological emergency departments. About 50% of all patients received or were prescribed at least one medication. The administration or prescription of medication was significantly higher in Turkish INT patients compared with German patients. There was no ethnic difference regarding medication in gynaecological cases. Almost all consultations were held in German. Language interpretation – usually by a non-professional interpreter (e.g. family member) – was used in one-third of the consultations with Turkish patients.

### Impact of gender and ethnicity on doctors' satisfaction with the course of treatment

The doctors' satisfaction with the course of treatment differed significantly in relation to the doctor's sex and professional experience. In the INT emergency departments, female doctors were less satisfied with the course of treatment than their male colleagues (10.8% vs. 14.5%, p < .05). Specialists and senior doctors more frequently rated their satisfaction with treatment as poor. These differences were less pronounced in the GYN clinics.

**Figure 1:**
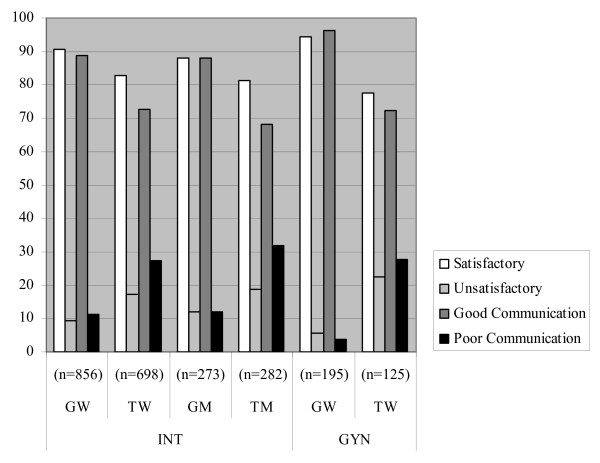
**Effect of patient sex and ethnicity on the satisfaction of doctors with the doctor-patient relationship (in %)**. GW = German women, GM= German men, TW = Turkish women, TM = Turkish men ^1) ^Kruskal-Wallis-Test and median test: p ≤ 0.01.

The doctors' satisfaction with the course of treatment was lower when attending Turkish patients than with German patients (Figure 1). Overall, 17% and 19% of doctors were dissatisfied with the course of treatment when attending Turkish women and men, respectively, compared to dissatisfaction rates of 9% and 12% with German women and men. The doctors experienced communication problems in one-third of their encounters with Turkish patients. Doctors' satisfaction with the course of treatment also showed sex-related variations: male doctors were more frequently dissatisfied than female doctors. The male patient/female doctor dyad (M/F) resulted in the highest proportion of treatment dissatisfaction (17.2% compared to 8.9% for F/F, 7.3% for F/M and 9.0% for M/M). A significant difference was only found within the German patient sample (p <= 0.001). Similar results were found for specified communication problems.

The main reasons stated as a cause for the doctors' dissatisfaction were the patients' "unwillingness to cooperate/communicate" and "inadequate communication competence (mostly based on lacking language skills"). These responses differed according to the patient's sex and ethnicity (see Table 6), but not according to the doctors' sex and professional experience. The main reason for doctors' dissatisfaction with German patients was "patient unwillingness to cooperate/communicate", whereas language-related communication problems dominated on the Turkish side. The second most frequent reason for dissatisfaction with treatment of German male patients was excessive alcohol consumption.

**Table 6:** Reasons for doctor's dissatisfaction with doctor-patient relationship

	Internal Medicine				Gynaecology	
Categories (%)	GW (n = 43)	TW (n = 25)	GM (n = 47)	TM (n = 32)	GW (n = 10)	TW (n = 13)

Patient unwillingness to cooperate/communicate	62.4	4.0	40.4	18.8	50.0	0
Patient alcohol abuse	7.0	0	27.7	6.3	0	0
Difficult patient	11.6	24.0	10.6	3.1	10.0	15.4
Communication problems	2.3	40.0	0	34.4	0	38.5
Inappropriate utilisation of emergency services	2.3	16.0	10.0	12.5	30.0	23.1
Too little time	4.7	0	0	3.1	0	0
Adequate treatment not possible	0	4.0	4.3	6.3	10.0	7.7
Psychosocial problems	0	0	2.1	0	0	0
Other reasons	9.3	12.0	4.3	15.6	0	15.4

GW = German women, GM = German men, TW = Turkish women, TM = Turkish men; Kruskal-Wallis test, Internal Medicine: p ≤ 0.01; Gynaecology: n.s.

Furthermore, the bivariate analysis showed a strong positive correlation between doctors' satisfaction and good communication (r = .69), and a weak negative correlation between the lack of urgency for emergency treatment as perceived by the doctors (r = -.12). These trends were observed in all subgroups.

### Multivariate analysis

Different models were tested by logistic regression analysis to determine the effects of various factors on the doctors' satisfaction with the course of treatment (see Table 7). The first model (M1) analysing patient age, sex and ethnicity showed that male sex (OR = 1.40) and Turkish ethnicity (OR = 2.61) increased the probability of doctors' dissatisfaction; this effect was stronger in GYN emergency departments (OR = 5.52) than in INT emergency departments. Model 2 (M2), which additionally evaluated communication problems and the need for emergency treatment as perceived by the doctor, showed the greatest impact through communication problems (OR = 5.28 in INT and 4.07 in GYN) and the lack of urgency for emergency treatment as perceived by the doctors (OR = 0.75 in INT and 0.71 in GYN). Consequently, a high perceived need for emergency treatment reduced the probability of doctors' dissatisfaction. The last model (M3) evaluated the following variables in a stepwise forward logistic regression analysis: patient age, sex and ethnicity, utilisation of interpreter, type of health insurance, time of presentation, means of transportation, duration of complaints, need for emergency treatment as perceived by the doctor, doctor's professional experience and sex, communication competence and length of medical history.

**Table 7:** Multivariate analysis of factors influencing doctors' satisfaction with doctor-patient relationship (logistic regression) using three models (M1, M2, M3)

Internal Medicine	M1^1)^			M2^1)^			M3^2)^		
	OR	95% CI		OR	95% CI		OR	95% CI	

Age	1.01***	1.01	1.02	1.00	0.99	1.01	-#-	-#-	-#-
Gender	1.40*	1.00	1.95	1.35	0.90	2.02	-#-	-#-	-#-
Ethnicity	2.61***	1.70	4.02	0.89	0.52	1.50	-#-	-#-	-#-
Gender * Ethnicity	0.78	0.45	1.36	.90	0.46	1.79	-#-	-#-	-#-
Communication competence	-*-	-*-	-*-	5.28***	4.34	6.43	4.48***	3.34	6.01
Need for emergency treatment ^3)^	-*-	-*-	-*-	0.75***	0.70	0.81	0.75***	0.67	0.83
Doctor's sex^4)^	-*-	-*-	-*-	-*-	-*-	-*-	0.50**	0.30	0.85
Health insurance^5)^	-*-	-*-	-*-	-*-	-*-	-*-	3.05**	1.31	7.11
Nagelkerke r^2^	.03			.41			.33		

Gynaecology	M1^1)^			M2^1)^			M3^2)^		

	OR	95% CI		OR	95% CI		OR	95% CI	

Age	1.03	0.99	1.05	1.01	0.97	1.04	-#-	-#-	-#-
Ethnicity	5.52***	2.50	12.17	1.87	0.71	4.94	-#-	-#-	-#-
Communication competence	-*-	-*-	-*-	4.07***	2.52	6.56	6.22***	2.84	13.62
Need for emergency treatment ^3)^	-*-	-*-	-*-	0.71***	0.60	0.84	0.63***	0.55	0.81
Duration of complaints	-*-	-*-	-*-	-*-	-*-	-*-	1.00	1.00	1.02
Nagelkerke r^2^	.13			.46			.50		

1) Dependent variable: satisfaction with doctor-patient relationship (reference category: "dissatisfied");

2) Stepwise logistic regression, only significant results are shown; ^3) ^Need for emergency treatment as perceived by the physician, ^4) ^Reference category: female; ^5) ^Reference category: health insurance not paid by the welfare office

OR – Odds Ratio; CI – Confidence Interval

* p ≤ .05; ** p ≤ .01; *** p ≤ .001; -*- not included in the model; -#- included, but non-significant

Communication problems (OR = 4.48), perceived lack of urgency for emergency treatment (OR = 0.75), doctor's sex (OR = 0.50) and health insurance coverage through welfare (OR = 3.05) were significant predictors of the doctors dissatisfaction in the INT subgroups. Communication problems (OR = 6.22) and the perceived lack of need for emergency treatment (OR = 0.63) were the most important predictors within the GYN subgroups.

## Discussion

The results of the present study demonstrate that the doctors' satisfaction with doctor-patient relationships correlate with sex and ethnicity; however, when communication problems are taken into account, the relevance of these factors disappears. Further analysis showed that socioeconomic differences between the groups (group 1: doctors satisfied with the course of treatment vs. group 2: doctors dissatisfied with the course of treatment) were minor and do not account for the presented results. So far, only few studies have explored the doctor-patient relationship and the impact of gender and ethnicity on it from the doctor's point of view [7,8]. Competence in the official language plays a major role in health care. In a systematic review, Anderson et al. [9] demonstrated that in multicultural healthcare systems the inability to communicate with healthcare providers creates barriers to access, undermines trust in the quality of medical care and decreases patient compliance (see also [10]). Since approximately 20% to 50% of Turkish immigrants living in Germany have only very basic German language skills, they are at risk to encounter these problems. The present study confirms that communication problems do indeed influence doctors' satisfaction with the doctor-patient relationship, and that they are the principal cause of doctors' dissatisfaction.

Patient-physician communication plays a crucial role for an improved patient health status and compliance as well as for the patients' satisfaction and can be regarded as a mediator of health care quality and patient safety ([11]). A recent systematic review [11] highlighted that communication intervention in the group of physicians yield significant improvements in the communication behaviour. Interventions physician are more likely to ask open-ended questions, to express empathy, to provide reassurance, and to provide information to patients. However, the findings regarding interventions in the group of patients are mixed, showing only partly an improvement in information providing behaviour or patient involvement.

Ethnic differences in communication patterns during doctor-patient encounters were demonstrated in different studies (see [12,13]). Immigrants from non-Western countries, in particular, faced shorter consultation times and a stronger hierarchy. The present study provides a rough analysis of communication problems. Comparison of the length of written medical histories did not reveal any significant differences between German and Turkish patients.

Gender dyads have been analysed in several studies [14-16]. Some investigators detected gender differences in verbal and non-verbal behaviour of physicians. For example, affective behaviour, such as empathy, emotional support, and encouragement, is more pronounced in female doctors. Differences in gender composition were also found in this study; interestingly, these were relevant only in the German sample. The reasons for this are speculative: the relatively high proportion of German males under the influence of alcohol was the second most common reason of doctors' dissatisfaction with the consultation.

Studies focusing on the role of interpreters in health care support the importance of language skills for adequate and high-quality health care. Language skills are crucial, but knowledge about cultural practices and specific needs of immigrants are equally important [17-19]. Fernandez et al. [20] showed a positive impact on the patient's perception of health care when doctors are able to communicate in the patient's native language. Non-professional interpreters (e.g. family members and hospital staff) can hardly fulfil these requirements [17]. Hultsjö & Hjelm [8], who studied nurses working in emergency departments, stress the impact of communication problems on the course of treatment and the need for the provision of qualified medical interpreters in health care. They concluded that intercultural communication skills are crucial for health care professionals. The effect of integrating interpreters into the medical system was also analysed by Harmsen et al. [17] in Netherlands. A randomised control study showed that an improved understanding between physicians and immigrants (Moroccan and Turkish) on both sides could be achieved through intercultural communication training. Especially in the context of emergency health care, proper understanding of the patient's complaints and needs plays a predominant role in the provision of satisfactory treatment.

So far, the German health care system has not adequately responded to immigrant health care needs and qualified interpreters are rarely provided by health care services. A recent study performed by the consumer protection agency of the states of North-Rhine/Westphalia and Rhineland-Palatinate [21] demonstrated that German hospitals provide insufficient support to patients with language barriers. The present study confirms these results. None of the participating hospitals provided professional interpretation. Little improvement of language-related communications barriers can be achieved under such circumstances.

Hudelson [18] identified the following sources of communication problems and misunderstandings: (1) misconceptions about the patient's health problem; (2) unrealistic expectations of the clinical encounter; and (3) differences in verbal and non-verbal communication styles. In emergency outpatient services, communication takes place under pressure due to the acute condition of the patient and the high work-related strain on the doctor; often accompanied by problems related to the organisational structure of emergences departments. Under these conditions, a higher frequency of misunderstandings is very likely, even in patients with a good command of the language (in this case, German). These misunderstandings can lower the satisfaction of both patients [7,18] and doctors, as the present study shows. Moreover, misunderstandings can also increase the risk of misdiagnosis and of inappropriate or unnecessary treatment [17]. Murphy [22] showed that inappropriate utilisation of emergency departments can have a negative impact on the doctor-patient-relationship, leading to conflicts and misunderstandings. Our results support these findings.

The data analysis of the present study does have several limitations. Due to the instruments used, only limited information existed regarding the doctor-patient relationship as perceived by the doctor and regarding potential influence variables, such as professional experience. Since working conditions and work strain in the two doctors' groups were comparable, we assumed that these variables would not have a major impact on the outcome variable. Several still unanswered questions should be investigated in more detail, including the effect of the doctor's ethnicity on his or her satisfaction with the consultation and the doctor-patient relationship as well as the effect of organisational circumstances on the doctors' satisfaction with the consultation. Further analysis should explore the role of ethnicity on communication patterns and the interactions between doctors and patients in more depth, integrating the impact on outcome of health care and using existing evidence-based frameworks, such as the Four Habits Model [23] for clarification and explanation.

## Conclusion

Language barriers in emergency departments have a negative impact on the doctor-patient relationship as well as on the doctors' satisfaction with the consultation. Essential prerequisites to ensure culturally competent health care have been developed and include the consideration and acceptance of cultural differences and the use of professional interpreters [7,18,24,25]. However, most health care facilities in Germany [5,24], including emergency departments, do not fulfil these requirements. To improve this situation the needs of patients and professionals ought to be considered and changes in the structure and management of emergency departments are required The following specific measure are recommended:

1. Patients:

• Provision of easily accessible, multilingual information about the health care system, and therein specific therapeutic and psychosocial facilities aimed at immigrants,

• Availability and accessibility of professional medical interpreters

2. Doctors

• Development and provision of intercultural communication courses for medical students

• Intercultural awareness training for professionals in emergency departments including: cultural differences in health seeking behaviour, complaints and symptom presentation, coping strategies, and cooperation with professional interpreters

• Awareness raising among emergency department doctors about their role as gate-keepers of care for immigrant patients

3. Emergency departments

• Ethnic monitoring by the emergency departments including: Recording of ethnicity as a quality indicator, recruitment of ethnic minority personnel, resourcing and management of emergency departments to meet the needs of immigrant patients (e.g. provision of multimedia and multilingual information, signposts, and interpreters).

## Competing interests

The author(s) declare that they have no competing interests.

## Authors' contributions

All authors contributed to the study. TB, ThB and MD made substantial contributions to study conception, design and data acquisition. BB performed the data analysis and drafted the manuscript. All authors read and approved the final manuscript

## Pre-publication history

The pre-publication history for this paper can be accessed here:



## References

[B1] Burchardi C, Angstwurm M, Endres S (2001). Diagnosespektrum in einer Internalen Notaufnahme [Spectrum of diagnoses in an internal medicine emergency unit]. Der Internist.

[B2] Sempere-Selva T, Peiro S, Sendra-Pina P, Martínez-Espín C, López-Aguilera I (2001). Inappropriate use of an accident and emergency department: Magnitude, associated factors, and reasons – an approach with explicit criteria. Annals of Emergency Medicine.

[B3] David M, Schwartau I, Pant HA, Borde T (2006). Emergency outpatient services in the city of Berlin: factors for appropriate use and predictors for hospital admission. European Journal of Emergency Medicine.

[B4] David M, Pette G, Kentenich H (1998). Unterschiedliche Inanspruchnahme einer gynäkologischen Notfallambulanz durch deutsche Patientinnen und Migrantinnen [Different utilization of a gynecological emergency department by German and immigrant patients]. Geburtshilfe und Frauenheilkunde.

[B5] Borde T, Braun T, David T (2003). Unterschiede in der Inanspruchnahme klinischer Notfallambulanzen durch deutsche Patienten/innen und Migranten/innen [Difference in utilization of emergency departments by German and immigrant patients]. Schlussbericht für das BMBF [Final report].

[B6] Spallek J, Kaatsch P, Spix C, Ulusoy N, Zeeb H, Razum O (2006). Namensbasierte Identifizierung von Fällen mit türkischer Herkunft im Kinderkrebsregister Mainz [Name-based identification of cases of Turkish origin in the childhood cancer registry in Mainz]. Gesundheitswesen.

[B7] Cooper L, Beach M, Johnson R, Inui T Delving below the surface. Understanding how race and ethnicity influence relationship in health care. J Gen Intern Med.

[B8] Hultsjö S, Hjelm K (2005). Immigrants in emergency care : Swedish health care staff's experiences. International Nursing Review.

[B9] Anderson LM, Scrimshaw SC, Fullilove MT, Fielding JE, Normand J (2003). Culturally Competent Healthcare Systems. A Systematic Review. Am J Prev Med.

[B10] Carrasquillo O, Orav J, Brennan TA, Burstin HR (1999). Impact of Language Barriers on Patient Satisfaction in an Emergency Department. J Gen Intern Med.

[B11] Rao JK, Anderson LA, Inui TS, Frankel RM (2007). Communication interventions make a difference in conversations between physicians and patient. Medical Care.

[B12] Johnson R, Roter D, Powe NR, Cooper LA (2004). Patient race/ethnicity and Quality of Patient-Physician Communication during medical visits. Am J Public Health.

[B13] Meeuwesen L, Harmsen J, Bernsen R, Bruijnzeels MA (2006). Do Dutch doctors communicate differently with immigrant patients than with Dutch patients. Soc Sci Med.

[B14] Hall JA, Roter DL (2002). Do patients talk differently to male and female physicians? A meta-analytic review. Patient education and counselling.

[B15] Roter DL, Hall JA, Aoki Y (2002). Physician Gender Effects in Medical Communication. A meta-analytic Review. JAMA.

[B16] Brink-Muinen A, Dulmen S, Messerli-Rohrbach V, Bensing J (2002). Do gender-dyads have different communication patterns? A comparative study in Western-European general practices. Patient education and counselling.

[B17] Harmsen H, Bernsen R, Meeuwsen L, Thomas S, Dorrenboom G, Pinto D (2005). The effect of educational intervention on intercultural communication: results of a randomised controlled trial. British Journal of General Practice.

[B18] Hudelson P (2005). Improving patient-provider communication: insights from interpreters. Family Practice.

[B19] Karliner L, Pérez-Stable E, Gildengorin G (2004). The language divide. The importance of training in the use of interpreters for outpatient practice. Gen Intern Med.

[B20] Fernandez A, Schillinger D, Grumbach K, Rosenthal A, Stewart AL, Wang F, Pérez-Stable E (2004). Physician Language Ability and Cultural Competence. An Exploratory Study of Communication with Spanish-speaking Patients. J Gen Intern Med.

[B21] Verbraucherzentrale Nordrhein-Westfalen [Consumer protection agency] Manko bei der Gesundheitsversorgung von Migranten. Krankenhäuser ignorieren Sprachdefizite [Deficits in health care of immigrants. Hospitals ignore language problems]. http://www.verbraucherzentrale-rlp.de/UNIQ114536359802295/link200294A.html.

[B22] Murphy A (1998). Inappropriate attenders at accident and emergency departments I: definition, incidence and reasons for attendance. Family Practice.

[B23] Stein T, Frankel RM, Krupat E (2005). Enhancing clinician communication skills in a large healthcare organization : A longitudinal case study. Patient Education and Counseling.

[B24] Borde T, David T, Kentenich H, David M, Borde T, Kentenich H (2000). Patientinnenorientierung im Kontext der kulturellen Vielfalt im Krankenhaus [Patient orientation in hospitals in the context of cultural diversity].

[B25] Ilkilic I (2007). Medizinethische Aspekte im Umgang mit muslimischen Patienten [Medical ethical aspects of culture in social interactions with Muslim patients]. Dtsch Med Wochenschr.

